# Effect of Quercetin on Preosteoblasts and Bone Defects

**DOI:** 10.2174/1874325000802010027

**Published:** 2008-03-10

**Authors:** R.W.K Wong, A.B.M Rabie

**Affiliations:** Biomedical and Tissue Engineering, University of Hong Kong, Prince Philip Dental Hospital, 34 Hospital Road, Sai Ying Pun, Hong Kong

**Keywords:** Bone repair, flavonol, phytoestrogen, quercetin.

## Abstract

Quercetin is a flavonol, also a phytoestrogen, available commonly in onion and apple. Our laboratory investigated its effect on MC3T3-E1 cells’ alkaline phosphatase activity *in vitro* and compared the amount of new bone produced by quercetin in collagen matrix to that produced by bone grafts and collagen matrix *in vivo*. Four bone defects, 5mm by 10mm were created in the parietal bone of 2 New Zealand White rabbits. In the experimental animal, 2 defects were grafted with quercetin solution mixed with collagen matrix. In the control animal, 2 defects were grafted with collagen matrix alone. Animals were killed on day 14 and the defects were dissected and prepared for histological qualitative assessment. Results showed that 10μM of quercetin increased alkaline phosphatase activity of MC3T3-E1 cells at 72 hours *in vitro* by 32%. In the experimental animal, there was new bone growing inside the bone defects. In conclusion, specific concentration of quercetin increased alkaline phosphatase activity of MC3T3-E1 cells *in vitro* and quercetin in collagen matrix has the effect of forming new bone across bone defects *in vivo*.

## INTRODUCTION

Discovery of chemicals that increase bone formation is needed for the treatment of osteoporosis or to improve bone healing after trauma or surgery. The decrease in serum estrogen after menopause is associated with bone loss and osteoporosis, and estrogen replacement therapy is considered to be effective in preventing bone loss (Turner ER 1994) [1]. It has been shown that estrogen enhances osteoblast differentiation and bone formation (Qu Bone 1998) [2] and the conditioned medium of estrogen-treated osteoblast cultures inhibits osteoclast development (Qu Bone 1999) [3]. Although there are concerns about the effectiveness of oestrogen replacement therapy in the prevention of osteoporosis, estrogen is one of the most important sex steroids for the maintenance of bone balance.

Phytoestrogens are plant-derived non-steroidal compounds that bind to estrogen receptors (ERs) and have estrogen-like activity (Branca PNS 2003) [4]. Phytoestrogens have attracted much attention among public and medical communities because of their potential beneficial role in prevention and treatment of cardiovascular diseases, osteoporosis, diabetes and obesity, menopausal symptoms, renal diseases and various cancers (Hathena AJCN 2002) [5] (Duncan BPRCEM 2003) [6].

Phytoestrogens are divided into three classes: isoflavones, coumestans and lignans. In addition, some flavonoids, such as flavonols, are also classed as phytoestrogens (Duncan BPRCEM 2003) [6]. Estrogen receptors exist as two subtypes, ERa and ERb, and osteoblasts express both receptors (Arts Endocrinology 1997) [7]. The interaction between flavonols and flavonoids with ERa and ERb is well documented (Kuiper Endocrinology 1998) [8]. Kuiper *et al*.  (Kuiper Endocrinology 1998) [8], who studied the estrogenic potency of several phytoestrogens, showed that quercetin have the capacity to bind both ER subtypes.

Quercetin, 3,3',4',5,7-Pentahydroxyflavone; 2-(3,4-Dihydroxyphenyl)-3,5,7-trihydroxy-4H-chromen-4-one, C_15_H_10_O_7_, molecular weight: 302.24, water solubility: <0.1 g/100 mL at 21°C, is one of the major phytoestrogens isolated from onion (200-600mg quercetin/kg onion) (Price JSFA 1997) [9], apple and grape. The average daily flavonoid intake in the occidental diet is around 23 mg, of which quercetin represents 60% to 75% (Hertog Lancet 1993) [10].

Kanno *et al*. (Kanno Toxicology 2004) [11] report the effects of phytoestrogens and environmental estrogens on osteoblast differentiation using MC3T3-E1 cells, a mouse calvaria osteoblast-like cell line. They increased alkaline phosphatase activity and enhanced bone mineralization in these cells. It is possible that quercetin also stimulates osteoblasts and increases the bone formation locally.

Therefore, if quercetin, this common constituent in fruits and vegetables, can be shown to increase bone forming activities in bone cells and improve healing in bone defects, it may be the long-sought-after safe and ideal agent for stimulation of bone formation and bone defect repair. The aims of this study were to examine quantitatively the effects of quercetin on MC3T3-E1 cells, a mouse calvaria preosteoblastic cell line and qualitatively the effect of quercetin on bone defects *in vivo*.

## MATERIALS AND METHODOLOGY

Cell culture: MC3T3-E1 mouse perosteoblasts (RIKEN, Japan) were cultured in Alpha Minimum Essential Medium MEMα (Invitrogen, USA) containing 10 vol% fetal bovine serum (Invitrogen, USA) and antibiotics penicillin G sodium 100 units/mL and streptomycin 100μL/mL (Invitrogen, USA) and incubated at 37°C in a 5% CO_2_/95% air humidified atmosphere (Declercq Biomaterials 2005) [12].

Control Group: Cells cultured without any intervention for different time intervals (24 hours, 48 hours and 72 hours and 14 days for mineralization).

Quercetin Group: Cells cultured with quercetin (Sigma, USA) of different concentrations (0.1μM, 1μM, 10μM and 100μM) for different time intervals (24 hours, 48 hours and 72 hours and 14 days for mineralization). 24 and 96-well tissue culture plates (Iwaki, Japan; 2.5×10^4^cells/cm^2^) were used. Each experimental condition was repeated 4 times. Cytoplasmic total protein assay: Total protein is an indication of the proliferative and biosynthetic capacities of bone cell cultures. Cells in 24 well plates were lysed and the cellular material was removed into 250μL of a buffer containing 10mM Tris HCl pH 7.5, 0.5mM MgCl_2_ and 0.1% Triton X-100. The cellular material was homogenized by two freeze-and-thaw cycles (Declercq Biomaterials 2004) [13]. The cellular protein concentration was determined by BCA protein assay kit (Pierce, IL, USA).

Biochemical detection of the alkaline phosphatase activity: The protocol of Declercq *et al*. (Declercq Biomaterials 2004) [13] was followed. After rinsing the monolayers in 24 well plates with Ringer solution, the cellular material was removed after lysis into 250 μL of a buffer containing 10 mM Tris HCl pH 7.5, 0.5 mM MgCl_2_ and 0.1% Triton X-100. The cellular material was homogenized by two freeze-and-thaw cycles. Alkaline phosphatase activity was determined with *p*-nitrophenylphosphate as the substrate. Sample volumes of 50 μL were added to 50 μL *p*-nitrophenylphosphate (4.34 mM) in 100 mM glycine, pH 10.3, 1 mM MgCl_2_ and incubated at 37°C for 30 min on a bench shaker. The enzymatic reaction was stopped by adding 50 μL of 1M NaOH. Enzyme activity was quantified by absorbance measurements at 405 nm and calculated according to a series of alkaline phosphatase standards. Alkaline phosphatase activity was expressed as unit of alkaline phosphatase/mg protein.

Statistical analysis: Data were analysed with a statistical analysis computer software (SPSS 15.0 for Windows©, SPSS Inc., Chicago, Illinois 60606). Data were performed with normality test (Kolmogorov-Smirnov test; *p*>0.05). The unpaired t-tests with Welch correction were used to compare with control and different concentrations of quercetin. Significance was set in advance at *p*<0.05.

*In vivo* qualitative study of quercetin in bone defect: The methodology and animal model used have been described previously (Wong BJOMS 2003) [14]. Four 10×5 mm^2^ full-thickness bone defects were created in the parietal bones of 2 New Zealand White rabbits from an inbred colony. The rabbits were 5 months old (adult stage) and weighed 3.5-4.0kg. The handling of the animals and the experimental protocol were approved by the Committee on the Use of Live Animals in Teaching and Research, the University of Hong Kong. In the experimental animal, 2 defects were grafted with collagen matrix carrier with quercetin solution. In the control animal, 2 defects were grafted with collagen matrix alone. The animals were premedicated 1 hour before surgery with oxytetracycline hydrochloride (200mg/mL, 30mg/kg body weight, Tetroxyla, Bimeda, Dublin, Ireland) and buprenorphine hydrochloride (0.3mL/kg body weight, Hypnorm, Janssen Pharmaceutical, Beerse, Belgium), supplemented with diazepam (5mg/mL, 1mg/kg body weight, Valium 10, Roche). In order to maintain the level of neuroleptanalgesia, increments of Hypnorm (0.1mL/kg) were given at 30-min intervals during the operation.

The surgical procedure consisted of the creation of two 10×5mm full-thickness (approximately 2mm) cranial defects, devoid of periosteum, using templates, in the parietal bones. The defects were produced using round stainless steel burs (1mm in diameter) on a low speed dental drill. Outlines of the defects were made initially by making holes of full thickness the parietal bone using a stainless steel wire template bent to the required size of the defect. The holes were joined to complete the process. During the cutting of bone, copious amount of sterile saline was used for irrigation and to minimize thermal damage to the tissues. In the experimental animal, the defects were filled with collagen matrix (purified absorbable fibrillar collagen, Collagen Matrix Inc, NJ, USA) with 0.2 mL quercetin solution (Sigma-Aldrich, MO, USA, dissolved in water for injection to the concentration of 100 mg/mL). The grafts were prepared 15 minutes before grafting. In the control animal, the defects were grafted with 0.02 g of collagen matrix (purified fibrillar collagen, Collagen Matrix, Inc NJ, USA) mixed with 0.2 mL water for injection.

All wounds were closed with interrupted 3/0 black silk sutures. No attempt was made to approximate the periosteum to prevent the barrier effect. Postoperatively, the rabbits were given oxytetracycline hydrochloride daily for 10 days and buprenorphine hydrochloride for 2 weeks.

Two weeks after surgery, the animals were killed with sodium pentobarbitone. Immediately upon death, defects and surrounding tissue were removed for histological preparation. Tissues were fixed in 10% neutral buffered formal saline solution, demineralized with K’s Decal Fluid (sodium formate/ formic acid), and finally double embedded in celloidin/ paraffin wax. Serial, 5-µm-thick sections of the whole defect were cut perpendicular to the long axis. The slides were stained with Periodic acid-Schiff stain which allowed easy identification of new bone.

## RESULTS

Cytoplasmic total protein assay: The total protein concentrations were shown in Table **1** and graphically in Fig. (**1**). There were no statistical differences between the experimental groups and the control groups at any concentration of quercetin in any time interval. The total protein concentrations in general increased between the time intervals from 24 to 48 hours.

Biochemical detection of the alkaline phosphatase activity: The alkaline phosphatase activities, expressed as the ratio with the total protein, were shown in Table **2** and graphically in Fig. (**2**). There were no statistical differences between the experimental groups and the control groups at any concentrations of quercetin in short time intervals (24 hours and 48 hours). At 72 hours, however, the activity is higher at in the experimental groups and was highly significant (*p*<0.001, 32% increase) at the concentration of 10 μM of quercetin.

*In vivo* study of quercetin: All animals remained in excellent health throughout the course of the experiment and recovered rapidly after operation. There was no evidence of side effects or infection in any of the animals. This was assessed by the in-house veterinary surgeons in the animal laboratory.

## DISCUSSION

Total protein is an indication of the proliferative and biosynthetic capacities of bone cell cultures. Results showed that quercetin did not have great effect on these capacities on the MC3T3-E1, however, it significantly increased the alkaline phosphatase activity in 72 hours by 32%. Showing that it had a stimulating effect on these preosteoblastic cells but the effect was only apparent at day three.

In this study unpaired t-test was used because it was necessary to compare each value of a particular time and concentration of extract with the control rather than the group difference in general. It is possible that at some concentrations and at some time frame there will be different in values between the experimental group and the control group but not the others, the result of this study showed this effect.

**Fig. (3):**
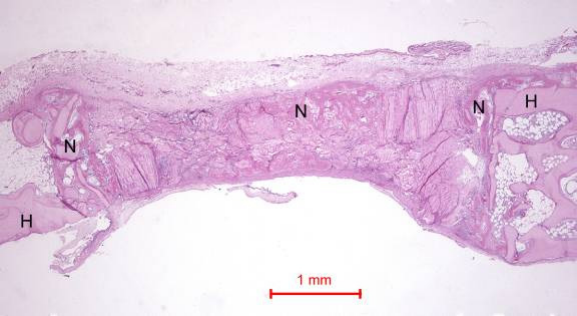
Photomicrograph of bony defect grafted with quercetin in collagen matrix on day 14. New bone (N) can be seen spanning across the defect. H = Host bone. Some collagen matrix (C) remained at the centre of the bony defect (Periodic acid-Schiff stain, original magnification ×40).

**Fig. (4):**
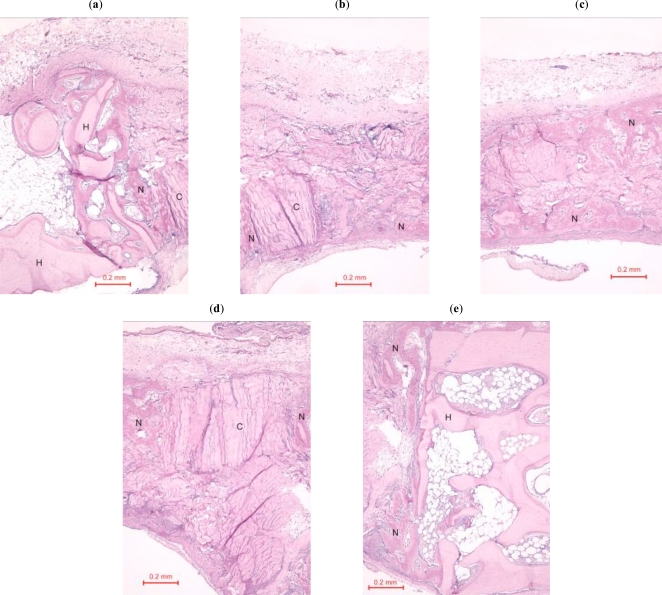
(**a**)-(**e**). Photomicrograph of bony defect grafted with quercetin in collagen matrix on day 14 (higher magnification). New bone (N) can be seen spanning across the defect and growing towards the collagen matrix where the quercetin was releasing out. H = Host bone. Some collagen matrix (C) remained at the centre of the bony defect (Periodic acid-Schiff stain, original magnification ×100).

In the rabbit grafted with quercetin in collagen matrix, new bone was formed at the host bone-graft interface and tended to grow across the defect (Fig. **3**). Integration of quercetin and collagen with the recipient bed was characterized by the presence of new bone. No cartilage was found. At higher magnification (Fig. **4**), new bone could be seen spanning across the defect and growing towards and amalgamating with the collagen matrix where the quercetin was releasing out.

**Fig. (5):**
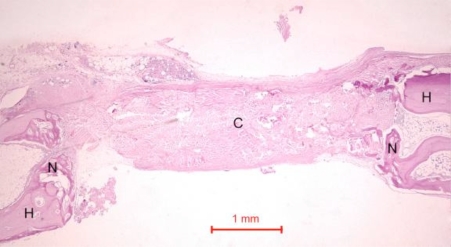
Photomicrograph of bony defect grafted with collagen matrix (positive control) in day 14. No bone could be seen across the defect except for a little new bone (N) near the ends of the host bone (H). Collagen matrix (C) remained across the bone defect (Periodic acid-Schiff stain, original magnification ×40).

In the control rabbit new bone was formed at the host bone-graft interface. Some collagen fibers were present at the centre of the defects (Fig. **5**). In both rabbits, the defect was healed, with fibrous tissue bridging across the defect.

These results demonstrated that quercetin significantly increased bone cell activities without significantly affecting their proliferation. As we also performed a qualitative assessment of quercetin solution in bone defect, for example, to check whether new bone will form or whether a severe inflammatory response will occur. These differences were compatible with the histological picture of the animal study. This is the first study that demonstrated the biological possibility of quercetin of causing new bone forming across the bone defect in this animal model in 14 days. Therefore this study discovered the potential of quercetin to be used as a local osteogenic agent. Further studies on various gene expressions on quercetin on osteoblasts are needed to confirm the actual stimulation mechanisms and to carry out a quantitative assessment of the amount of new bone formation of this extract compared with control with a greater sample size.

Prouilleta *et al.* (Prouilleta BP 2004) [15] showed that quercetin in the range of 1-50 mM, increase the activity of ALP in MG-63 human osteoblasts without any significant cytotoxic effect on the cells. The maximal stimulatory effect on ALP was observed after 48 hours of treatment with 50 mM quercetin. They also demonstrated that cycloheximide, a well known protein synthesis inhibitor, prevented the quercetin-induced ALP activation, indicating that de novo protein synthesis is essential for this response. Moreover, an activation of the ERK pathway is required for the observed phenomenon, because of the markedly reduction of the enhancing action of quercetin by MEK inhibitor PD 98059. The fact that ICI 182780, an antagonist of the ER, also prevents the quercetin-induced increase in ALP activity, indicates that this receptor is directly involved in the effect of quercetin. This study showed quercetin increased the ALP activity in MC3T3-E1 cells at the concentration of 10μM at 72 hours, showing the action of quercetin on different osteoblastic cell lines may be different. Further research is needed to compare the effect of quercetin on different osteoblastic cell lines under the same conditions.

Recently, the effects of quercetin on the differentiation and proliferation of human adipose tissue-derived stromal cells (hADSC) were determined (Kim BP 2006) [16]. Quercetin was found to increase osteogenic differentiation in a dose-dependent manner. Quercetin pretreatment administered prior to the induction of differentiation also exerted stimulatory effects on the osteogenic differentiation of hADSC. RT-PCR and real time PCR analysis showed that quercetin treatment induced an increase in the expression of osteopontin, BMP2, alkaline phosphatase and Runx2. Quercetin inhibited the proliferation of hADSC, but did not affect their survival. The pretreatment of quercetin increased ERK phosphorylation during osteogenic differentiation, although it did not increase ERK activity in control culture condition. ICI182780, a specific estrogen receptor antagonist, failed to inhibit the effects of quercetin on osteogenic differentiation. These findings indicated that quercetin enhances osteogenic differentiation *via *an independent mechanism from ER activation.

Further research on various gene expressions in osteoblasts, mesenchymal cells and endothelial cells during the early healing of quercetin in collagen matrix is needed to confirm the mechanism of its osteogenic effect.

This study has considerable significance as quercetin is a commonly used health supplement. It gained interests in research by its multiple health maintenance effects.

This is the first study that demonstrated quercetin local osteogenic effect and its possibility in causing bone formation at the centre of the bone defect, which was rare with other osteogenic agents (Wong BJOMS 2003) [14] (Wong JOMS 2006) [17] (Wong Biomaterials 2007) [18] (Wong OC 2007) [19] (Wong BJMRA in press) [20]. The limitations of this study are: Firstly, this study is looking at the histological picture qualitatively about the effect of grafting quercetin in bone defects, a quantitative assessment is needed to compare the amount of bone formed between the experimental and control group with a bigger sample size to show whether quercetin increases in bone formation. Secondly, there are concerns about the effectiveness of oestrogen replacement therapy in the prevention of osteoporosis, therefore, further studies are needed to determine the systemic effect of quercetin on bone metabolism on bone before clinical trials for its use in the prevention of osteoporosis to be considered.

## CONCLUSION

Specific concentration of quercetin increased alkaline phosphatase activity of MC3T3-E1 cells *in vitro*. This study also discovered the potential of quercetin to be used as a local osteogenic agent *in vivo*. Further research is needed to optimize its use and to gain further understanding on its bone forming mechanism.

## Figures and Tables

**Fig. (1):**
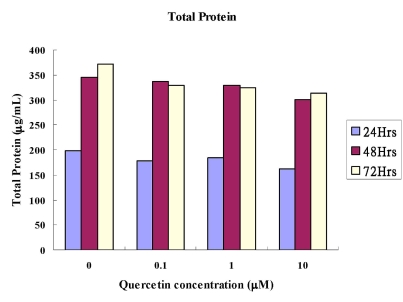
Comparison of amounts of the total protein (µg/mL) of quercetin on MC3T3-E1 cells.

**Fig. (2):**
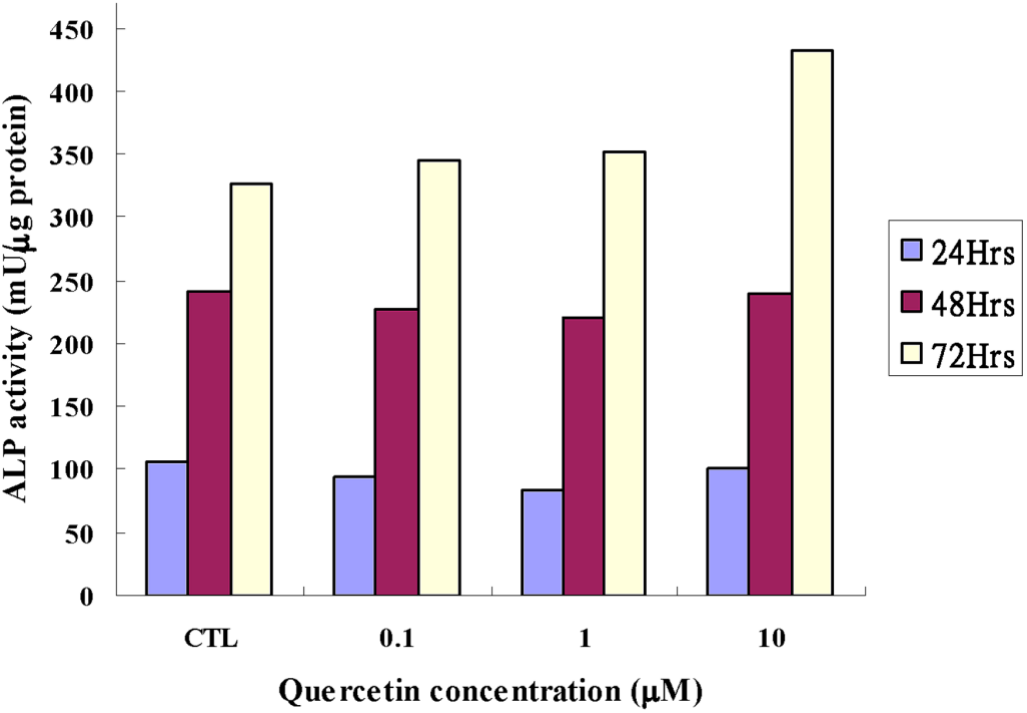
Comparison of alkaline phosphatase activity (mU/µg protein) of quercetin on MC3T3-E1 cells.

**Table 1:** Comparison of Amounts of the Total Protein (µg/mL) of Quercetin on MC3T3-E1 Cells (Mean±SD). *p*-Value was Derived from Unpaired T Test Comparing the Experimental Group with the Control

Concentration of Quercetin (µM)	0 (Control)	0.1	1	10
Total Protein, 24 hours (µg/mL) *p*-value	198.69±41.26 -	177.27±28.62 NS	184.83±25.16 NS	162.15±30.90 NS
Total Protein, 48 hours (µg/mL) *p*-value	344.86±37.38 -	337.30±23.64 NS	329.74±12.00 NS	300.76±14.48 NS
Total Protein, 72 hours (µg/mL) *p*-value	371.32±62.33 -	328.48±12.60 NS	324.70±19.52 NS	313.36±11.18 NS

NS: *p*> or =0.05, not statistical significant.

**Table 2:** The Alkaline Phosphatase Activity (mU/µg Protein) of Quercetin on MC3T3-E1 Cells (MEAN±SD). *p*-Value was Derived from Unpaired T Test Comparing the Experimental Group with the Control.

Concentration of Quercetin (µM)	0 (Control)	0.001	0.005	0.01
ALP, 24 hours (mU/µg protein) *p*-value	105.50±12.78 -	94.32±15.58 NS	83.68±8.21 NS	100.38±12.59 NS
ALP, 48 hours (mU/µg protein) *p*-value	240.17±24.19 -	227.95±15.00 NS	220.32±19.42 NS	239.53±25.82 NS
ALP, 72 hours (mU/µg protein) *p*-value	326.94±25.91 -	345.07±25.38 NS	352.02±26.12 NS	431.87±26.82 *p*<0.001

NS: *p*> or =0.05, not statistical significant.
